# Placental Histopathology in COVID-19-Positive Mothers

**DOI:** 10.4014/jmb.2206.06056

**Published:** 2022-08-11

**Authors:** Nikita Sherwani, Neha Singh, Arvind Neral, Jyoti Jaiswal, Tripti Nagaria, Onkar Khandwal

**Affiliations:** 1 Virology Lab, Department of Microbiology, Pt. JNM Medical College, Raipur, Chhattisgarh 492001, India; 2 Department of Pathology, Pt. JNM Medical College, Raipur, Chhattisgarh 492001, India; 3 Department of Obstetrics and Gynaecology, Pt. JNM Medical College, Raipur 492001, Chhattisgarh, India; 4 Department of Paediatrics, Pt. JNM Medical College, Raipur 492001, Chhattisgarh, India

**Keywords:** Pregnancy, COVID-19, placenta, pathology, SARS-CoV-2, RT-PCR

## Abstract

The placenta is a captivating multifunctional organ of fetal origin and plays an essential role during pregnancy by intimately connecting mother and baby. This study explicates placental pathology and information about 25 placentas collected from the mothers infected with novel coronavirus (SARS-COV-2). So far, congenital transmission of SARS-CoV-2 seems to be remarkably uncommon in spite of many cases of COVID-19 during pregnancy. Out of the 25 placental tissue samples collected, none has shown gene expression of SARS-CoV-2 when confirmed by RT-PCR. At the same time, nasal and throat swab samples collected from newborns of SARS-CoV-2-positive mothers correspondingly tested negative by RT-PCR. The shielding properties of placental barriers against viral infections from mothers to newborns remains a mystery. Major histopathological findings have been recorded as choriodecidual tissue with necrosis, intramural fibrin deposition, chorionic villi with fibrosis, and calcification. Moreover, although recent findings are insufficient to prove direct placental transmission of COVID-19, the abundance of angiotensin-converting enzymes-2 (ACE-2) on the placental surface could potentially contribute to unpleasant outcomes during pregnancy as SARSCoV-2 gains access to human cells via ACE-2. Finally, the significance of these findings is vague and needs further study.

## Introduction

With the recent pandemic of novel coronavirus (SARS-CoV-2), hospitals had an influx of COVID-19-positive mothers who were preparing for delivery. Due to the worldwide spread of COVID-19, information on adverse pregnancy outcomes has emerged in the literature, such as preeclampsia, preterm delivery, miscarriage, intrauterine fetal demise, and neonatal death [1]. The main binding receptor for SARS-CoV-2 on host cell is angiotensin-converting enzyme 2 (ACE2) receptor. ACE2 is expressed in the placenta [2] and is found in the syncytiotrophoblast, cytotrophoblast, endothelium, and vascular smooth muscle from both primary and secondary villi [3]. Innovative methodical research found evidence that ACE2 is expressed in various gynecologic organs such as the ovary, uterus, and vagina. Overall, ACE2 expression has been seen in numerous tissues in direct relation with developing pregnancies that could be associated with adverse maternal-fetal outcomes. Congenital infection can be challenging to characterize since pathogen detection usually requires specific methods. The placenta represents a highly specialized organ that maintains optimal environment for fetal development. Placental evaluation after delivery provides useful information such as the identification of disease processes in the mother or infant that requires diagnoses to deliver a specific explanation for an adverse outcome related to disease [4]. It is well recognized that analysis of the placental histopathological changes can provide valuable information, considering that a variety of pathological agents, counting infectious ones, are associated with characteristic morphological findings [5]. The intention of present research work was to evaluate a successive series of placentas delivered at our institution from COVID-19-positive mothers [6].

## Materials and Methods

Twenty-five placentas were collected by the Department of Gynaecology and Obstetrics (Pt. JNM Medical College, Raipur, Chhattisgarh, India) at the time of delivery, from SARS-CoV-2-infected women during the period of January 2021 to June 2021. Placental samples were then submitted to the Department of Pathology at the same institution for further analysis. Due to the infectious nature of the tissue, fixation for 48 h was performed prior to dissection. Typical sections were fixed in formalin, processed into paraffin blocks, and stained with the usual hematoxylin and eosin stain. Clinical history and other information related to the pregnant women were recorded at the time of delivery for RT-PCR and placental tissues were submitted at the Virology Lab, Pt. JNMMC. Testing was performed using standard protocols. Nasopharyngeal and oropharyngeal swabs were collected from expectant mothers at the time of delivery for RT-PCR. Nasopharyngeal and oropharyngeal swabs from newborns were collected within 0-12 h of delivery. All mothers and health workers were wearing full-protective gear at the time of delivery inside the delivery room. Viral RNA was extracted from placental tissues as well as respiratory samples using a predefined kit (Himedia SARS-CoV-2 PCR Viral RNA Purification Kit, India). All samples were processed and tested by using a qRT-PCR kit (Quantiplus Multiplex COVID-19 Detection, V2.0, HUWELL, India) approved by ICMR, India. Test results were confirmed by pre-designed primers for E gene and N gene. The primer sequence for N gene was GTTTGGTGGACCCTCAGATT GGTGAACCAAGACGCAGTAT HEXTGG GTAAACCTTGGGGCCGACGTTGT-BHQ (WT) and for E gene was CENR: ATCGAAGCGCAGTAAGGATGCENP: FAMTGCTTTCGTGGTATTCTTGCTAGTTACACTBHQCENF:CGGAAGAGACAGGTACGTTAATAG.

## Results and Discussion

All expectant mothers, even if asymptomatic, were tested and confirmed positive for SARS-CoV-2 via RT-PCR assay at the time of delivery. Nasopharyngeal and oropharyngeal swabs were collected from the mothers at the time of delivery for confirmation of COVID-19 positivity, whereas all newborn infants were shown to be negative by RT-PCR test within 0-12 h of birth. Clinical history and other information on the pregnant mothers were recorded at the time of delivery. Admitted mothers, positive for SARS-CoV-2, were between 18 to 33 years of age. Out of 25 deliveries, 11 women delivered vaginally and 14 delivered by caesarean section. All the mothers initiated breastfeeding immediately after birth and in full-protective gear. Out of the 25 mothers, 15 were asymptomatic and 10 were having mild to moderate symptoms like cough, fever, loss of smell, myalgia similar to non-pregnant women, and placental weight was recorded as ranging from 410-550 gm, with the shape of oval to discoid. RT-PCR of all placental tissue showed negative results for both gene E (envelope) and N (nucleocapsid), respectively (Table 1). For histopathology, placental tissues were fixed in formalin, processed into paraffin blocks, and stained with the usual hematoxylin and eosin stain. Microscopic examination of placental tissues was done in order to study the manifestation of the disease. Predominantly, results showed choriodecidual tissue with necrosis, chorionic villi with fibrosis, intramural fibrin deposition, and calcification in most of the cases (Table 2). Fibrin deposition is frequent during pregnancy as placental fibrinoids, which are extracellular depositions, can be found at all stages of pregnancy in almost all normal and pathological placenta. In our case, mild-to-moderate deposition of fibrin was observed, as was Massive Perivillous Fibrin Deposition (MPDD), an indication of infrequent placental lesion which has been associated with placental inflammation and an increased risk of adverse pregnancy outcomes such as fetal growth restriction (FGR) [7]. Prevalence of plasma cell deciduitis in the placenta has also been observed to be significantly higher in cases with MPFD [8]. Placental calcification and deposition of calcium-phosphate minerals in placenta tissue is common and can be seen in patients with and without placental diseases [9]. In our findings, one patient associated with marked calcification on the placenta also had a history of hypertension, with preterm labor, preeclampsia, and meconium. Highly calcified, grade III placentas often prompt expedited delivery and have been associated with a higher risk of adverse pregnancy outcome [10]. Calcification is normally observed in the placenta, but the incidence and interaction with acute clinical outcomes remains unclear. Clinical research on placental calcification is limited and discordant. Grade II is associated with lower birth weight and grade III with growth retardation, but neither is associated with poor prenatal outcome, maternal hypertension, fetal distress, or prenatal asphyxia [11]. It would be difficult to relate COVID-19 disease and marked placental calcification. Five placental tissues, out of 25, from coronavirus-positive mothers were recorded as having choriodecidual tissues with necrosis. The tissues were also noted to have other medical complications such as PROM, severe preeclampsia, preterm labor, hypertension, and meconium. Necrosis of placental membranes is a histologic lesion of unclear pathogenesis that has been reported in placentas from preeclampsia, preterm premature rupture of membranes, and preterm abruption [12]. Choriodecidual tissues displayed a different pro-inflammatory secretion pattern of cytokines and chemokines. The leading cause of preterm labor and rupture of membranes involves an inflammatory response involving increased production of cytokines as confirmed by studies [13]. Two placental samples were recorded with choriodecidual tissues with fibrosis. Though there are few reports on pulmonary fibrosis and COVID-19 disease, there is mounting evidence that fibrotic changes and interstitial lung abnormalities may result from COVID‐19 infection in some cases, but it is unclear that COVID-19 is promoting fibrosis during pregnancy as well, and further studies are needed to conclude whether the fibrosis is stable or progressive during COVID-19 infections [14]. SARS-CoV-2 infection has been shown to involve a wide range of organs and tissues, leading to a complex pattern of clinical conditions. Looking into current scenario, the histopathological evaluation of placentas delivered by women with SARS-CoV-2 infection did not confirm any distinct hallmarks. The inflammatory activation, lesions and marked calcification, and increased thrombotic risk described in patients with COVID-19 make the placenta a potential target of pathophysiological phenomena which could adversely affect pregnancy outcomes [15]. Exceptionally little is currently known about the effects of COVID-19 on the human placenta and neonate birth outcome. More research is urgently needed to confirm the outcomes of COVID‐19 infection during pregnancy.

In pregnant women with SARS-CoV-2 infection, a considerable proportion of placentas showed by histopathological report to have chorionic villi with calcification and intramural fibrin deposition. However, in some cases choriodecidual tissue with necrosis indicates inflammation and histologic lesion on placental tissue which could be associated with any previous pathogenesis. Hence, in the future, multicenter prospective studies are desirable to correlate these placental consequences during pregnancy with COVID-19.

## Figures and Tables

**Fig. 1:**
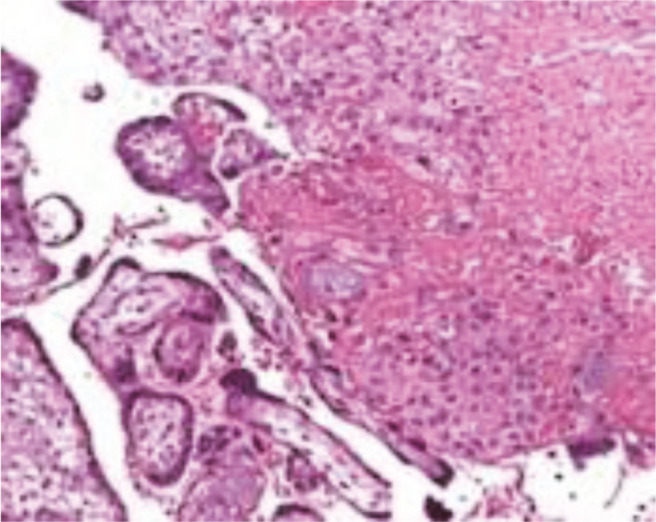
Choriodecidual tissue with necrotic villi and acute inflammation (H & E x400).

**Fig. 2:**
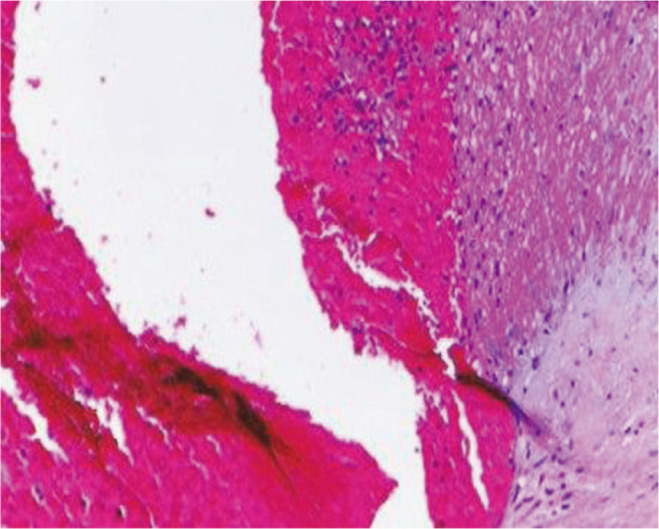
Intramural fibrin deposition (H & E x400).

**Fig. 3:**
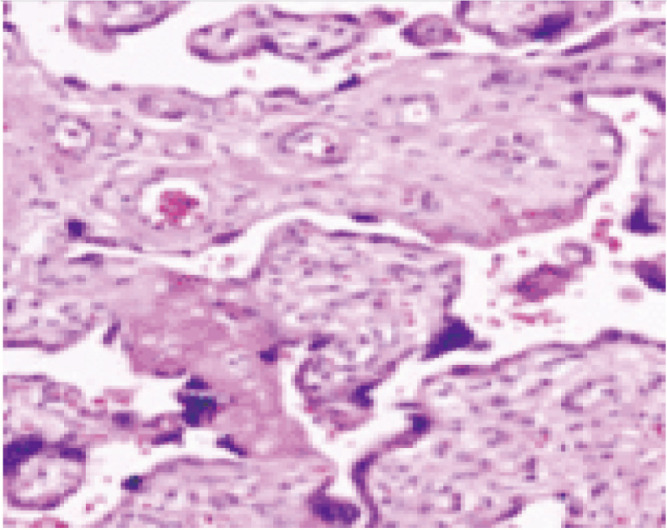
Chorionic villi showing fibroid changes (H & E x400).

**Table 1:** Clinical information.

Cases	Maternal age	GA	Neonate birthweight (Kgs)	Delivery	History	COVID related symptoms	Type of feeding	Placenta weight (g)	Shape of placenta	Size of placenta	RT-PCR of Naso & oropharyngeal swabs of pregnant mothers (at the time of delivery)	RT-PCR of Naso & oropharyngeal swabs of New-born (at the time of birth within 0-12 h)	RT-PCR of Placental tissue collected at the time of delivery
1	28	38w2d	2.7	LSCS	NA	No	BF	500	Discoid	15*18	+ve	-ve	-ve
2	23	37w2d	3	LSCS	NA	Yes	BF	500	NA	18*20	+ve	-ve	-ve
3	19	28w4d	2.5	LSCS	Preterm labor, preeclampsia, HTN, Meconium	Yes	BF	440	NA	15*16	+ve	-ve	-ve
4	23	35w1d	1.9	LSCS	NA	No	BF	450	Discoid	16*18	+ve	-ve	-ve
5	22	38w4d	3	LSCS	PPH, Uterineatony	No	BF	420	Discoid	18*16	+ve	-ve	-ve
6	33	39w3d	3.2	LSCS	Hypothyroidism	No	BF	450	Discoid	18*17	+ve	-ve	-ve
7	29	37w4d	2.4	LSCS	Meconium,	No	BF	430	NA	17*16	+ve	-ve	-ve
8	24	37w4d	2.5	VD	PROM	Yes	BF	450	NA	NA	+ve	-ve	-ve
9	20	36w9d	3	LSCS	Severe preeclampsia, HTN	No	BF	410	NA	NA	+ve	-ve	-ve
10	22	38w0d	3.2	VD	NA		BF	450	NA	NA	+ve	-ve	-ve
11	22	39w7d	2.2	VD	NA	No	BF	500	Oval	16*17	+ve	-ve	-ve
12	18	37e8d	2.1	LSCS	Meconium	No	BF	450	Oval		+ve	-ve	-ve
13	20	40w5d	2.6	VD	Jaundice with Sickle cell trait	Yes	BF	500	NA	NA	+ve	-ve	-ve
14	22	36w0d	1.4	VD	Meconium	No	BF	500	Oval	NA	+ve	-ve	-ve
15	23	36w2d	3.3	LSCS	Hypothyroidism	No	BF	500	Oval	NA	+ve	-ve	-ve
16	22	27w2d	2.7	LSCS	Preterm labor, PROM, Meconium	No	BF	420	Oval	NA	+ve	-ve	-ve
17	22	38w0d	2.5	LSCS	NA	No	BF	480	Oval	NA	+ve	-ve	-ve
18	22	39w3d	2.7	VD	With Sickle cell trait	No	BF	510	NA	NA	+ve	-ve	-ve
19	20	36w1d	2.9	LSCS	Twin with Hypothyroidism	No	BF	470	NA	NA	+ve	-ve	-ve
20	24	33w2d	3.1	VD	NA	Yes	BF	440	Oval		+ve	-ve	-ve
21	24	39w5d	2.6	VD	With Sickle cell trait	Yes	BF	520	NA	16*17	+ve	-ve	-ve
22	21	38w0d	2.8	VD	NA	Yes	BF	550	NA	19*15	+ve	-ve	-ve
23	28	40w3d	2.8	VD	NA	Yes	BF	500	Oval	NA	+ve	-ve	-ve
24	23	21w3d	3	LSCS	Preterm labor, Meconium	Yes	BF	480	Oval	NA	+ve	-ve	-ve
25	21	36w0d	3	VD	Single loop of cord around neck	Yes	BF	470	Discoid	18*17	+ve	-ve	-ve

Abbreviation: VD: vaginal delivery; w: weeks; d: days; GA: gestational age; LSCS: lower segment cesarean section; BF: breastfeeding; NA: not applicable; g: gram; HTN: hypertension; PPH: Postpartum hemorrhage; -ve: negative; RT-PCR:

**Table 2:** Observations of histopathological examination of placentas.

Cases	Histopathology of placenta
1	Choriodecidual tissue calcification
2	Choriodecidual tissue, intramural fibrin deposition
3	Choriodecidual tissue with necrosis, marked calcification
4	Calcification
5	Choriodecidual tissue, fibrosis, calcification
6	Choriodecidual tissue, fibrosis
7	Choriodecidual tissue with calcification
8	Intramural fibrin deposition
9	Choriodecidual tissuewith necrosis, intramural fibrin deposition, thicken bacterial vaginosis
10	None
11	Autolysed
12	Intramural fibrin deposition
13	Choriodecidual tissue with necrosis, calcification
14	Calcification
15	Chorionic villi with calcification
16	Choriodecidual tissue with necrosis, calcification
17	None
18	Chorionic villi
19	Chorionic villi with calcification
20	None
21	None
22	None
23	None
24	Choriodecidual tissue with necrosis, calcification
25	None
